# 4-(9-Anthr­yl)-1-(1-naphth­yl)spiro­[azetidine-3,9′-xanthen]-2-one *n*-hexane hemisolvate

**DOI:** 10.1107/S1600536808039305

**Published:** 2008-11-29

**Authors:** Mehmet Akkurt, Aliasghar Jarrahpour, Edris Ebrahimi, Mustafa Gençaslan, Orhan Büyükgüngör

**Affiliations:** a Department of Physics, Faculty of Arts and Sciences, Erciyes University, 38039 Kayseri, Turkey; b Department of Chemistry, College of Sciences, Shiraz University, 71454 Shiraz, Iran; c Department of Physics, Faculty of Arts and Sciences, Ondokuz Mayıs University, 55139 Samsun, Turkey

## Abstract

In the title compound, C_39_H_25_NO_2_·0.5C_6_H_14_, the β-lactam ring is nearly planar [maximum deviation of 0.012 (2) Å from the mean plane] and makes dihedral angles of 36.41 (13), 88.87 (13) and 54.16 (12)°, respectively, with the naphthalene, xanthene and anthracene ring systems. The mol­ecular conformation is stabilized by intra­molecular C—H⋯O and C—H⋯N contacts. The complete solvent mol­ecule is generated by inversion. In the crystal structure, mol­ecules are linked to each other by C—H⋯π inter­actions.

## Related literature

For general background on β-lactam anti­biotics, see: Bose *et al.* (2000 ▶); Banik & Becker (2000 ▶); Jarrahpour & Khalili (2007 ▶); Chincholkar *et al.* (2007 ▶); Cremonesi *et al.* (2004 ▶); Macias *et al.* (2004 ▶); Banik *et al.* (2003 ▶, 2004 ▶). For the crystal structures of related compounds, see: Akkurt *et al.* (2006 ▶, 2007 ▶, 2008 ▶); Pınar *et al.* (2006 ▶). For geometric analysis, see: Cremer & Pople (1975 ▶).
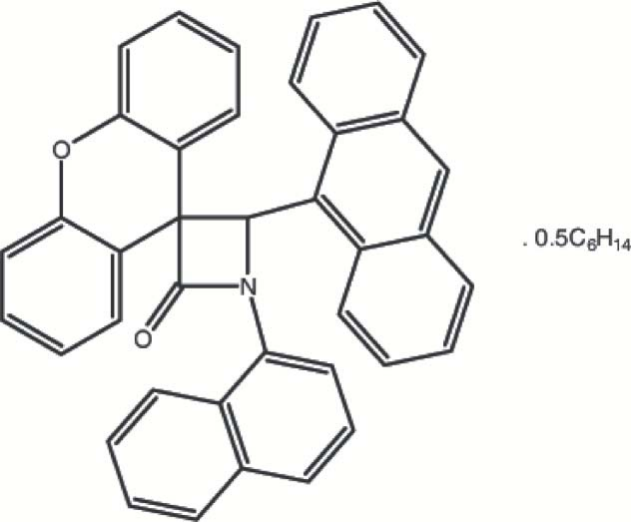


## Experimental

### 

#### Crystal data

C_39_H_25_NO_2_·0.5C_6_H_14_
*M* *_r_* = 582.69
Monoclinic, 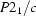

*a* = 12.0788 (5) Å
*b* = 14.1379 (5) Å
*c* = 18.6133 (8) Å
β = 107.216 (3)°
*V* = 3036.2 (2) Å^3^
*Z* = 4
Mo *K*α radiation
μ = 0.08 mm^−1^
*T* = 295 (2) K
0.55 × 0.38 × 0.26 mm

#### Data collection

Stoe IPDS2 diffractometer
Absorption correction: integration (*X-RED32*; Stoe & Cie, 2002 ▶) *T*_min_ = 0.959, *T*_max_ = 0.980
18377 measured reflections
6897 independent reflections
3841 reflections with *I* > 2σ(*I*)
*R*_int_ = 0.077

#### Refinement

*R*[*F* ^2^ > 2σ(*F* ^2^)] = 0.063
*wR*(*F* ^2^) = 0.191
*S* = 0.97
6897 reflections
406 parameters
4 restraints
H-atom parameters constrained
Δρ_max_ = 0.38 e Å^−3^
Δρ_min_ = −0.34 e Å^−3^

### 

Data collection: *X-AREA* (Stoe & Cie, 2002 ▶); cell refinement: *X-AREA*; data reduction: *X-RED32* (Stoe & Cie, 2002 ▶); program(s) used to solve structure: *SIR97* (Altomare *et al.*, 1999 ▶); program(s) used to refine structure: *SHELXL97* (Sheldrick, 2008 ▶); molecular graphics: *ORTEP-3* (Farrugia, 1997 ▶); software used to prepare material for publication: *WinGX* (Farrugia, 1999 ▶).

## Supplementary Material

Crystal structure: contains datablocks global, I. DOI: 10.1107/S1600536808039305/hb2859sup1.cif

Structure factors: contains datablocks I. DOI: 10.1107/S1600536808039305/hb2859Isup2.hkl

Additional supplementary materials:  crystallographic information; 3D view; checkCIF report

## Figures and Tables

**Table 1:** Hydrogen-bond geometry (Å, °)

*D*—H⋯*A*	*D*—H	H⋯*A*	*D*⋯*A*	*D*—H⋯*A*
C6—H6⋯N1	0.93	2.26	2.919 (3)	128
C38—H38⋯O1	0.93	2.41	3.074 (3)	128
C9—H9⋯*Cg*3^i^	0.93	2.60	3.471 (3)	156
C40—H40*B*⋯*Cg*1	0.97	2.82	3.572 (6)	135
C41—H41*A*⋯*Cg*2^ii^	0.97	2.85	3.728 (7)	150

Symmetry codes: (i) 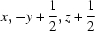; (ii) 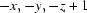. *Cg*1, *Cg*2 and *Cg*3 are the centroids of the C4/C5/C10–C12/C17, C5–C10 and C30–C34/C39 rings, respectively.

**Fig. 1.**
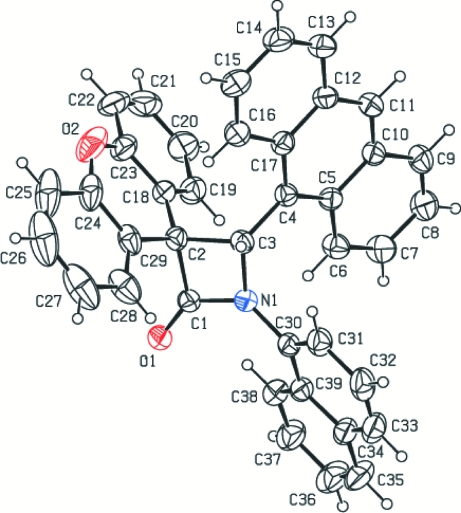
The molecular structure of (I): displacement ellipsoids for non-H atoms are drawn at the 30% probability level. The solvent molecule have been omitted for clarity.

**Fig. 2.**
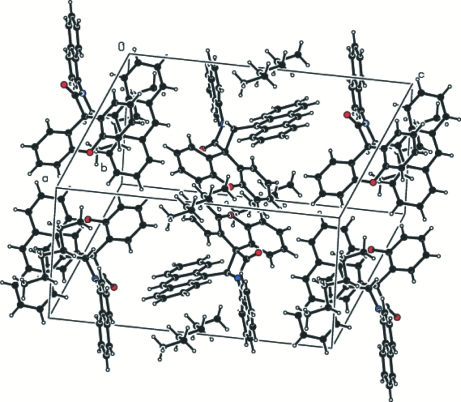
View of the packing diagram of (I).

## supplementary crystallographic information

### Comment

The role of β-lactam antibiotics is well known and since 1945, these have saved many lives (Bose *et al.*, 2000; Banik & Becker, 2000). Several syntheses of spiro-β-lactams are available in the literature (Jarrahpour & Khalili, 2007; Chincholkar *et al.*, 2007; Pınar *et al.*, 2006; Akkurt *et al.*, 2008). Many of them have employed the [2 + 2] cyclocondensation of ketenes and imines (Cremonesi *et al.*, 2004; Macias *et al.*, 2004), better known as the Staudinger reaction. The synthesis of novel anticancer β-lactams starting from imines, with pendent polyaromatic substituents have been reported (Banik *et al.*, 2003; Banik *et al.*, 2004).

In the title compound (I) (Fig. 1), the values of the geometric parameters of the β-lactam moiety (C1–C3/N1) are comparable with the values in our previously papers with the same unit (Akkurt *et al.*, 2008; Akkurt *et al.*, 2007; Pınar *et al.*, 2006; Akkurt *et al.*, 2006).

The β-lactam unit in(I) is nearly planar, with a maximum deviation of 0.012 (2) Å from the mean plane. This planarity is mainly due to the *sp*^2^ states of atoms C1 and N1. Atom O1 lies almost in the β-lactam plane, with a deviation of -0.029 (2) Å.

The dihedral angle between the naphthalene ring C30—C39 attached at N1 and the β-lactam is 36.41 (13)°.

In the xanthenering system, attached at C2, the benzene rings (C18–C23) and (C24–C29) are almost planar, forming a dihedral angle of 14.53 (17)° with each other. Its central ring, O2/C23/C18/C2/C29/C24, is not planar, with puckering parameters: Q_T _= 0.187 (3) Å, θ = 84.0 (9)° and φ = 178.8 (10)° (Cremer & Pople, 1975). The mean plane of the xanthene ring system forms the dihedral angles of 88.87 (13)°, and 54.96 (8)°, with the β-lactam ring and the naphthalene ring, respectively.

The anthracene ring system, attached at C3, is almost planar, with maximum deviations of-0.081 (2) Å for C4, 0.062 (3) Å for C6 and, 0.061 (3) Å for C7, makes dihedral angle of 54.16 (12)°, 82.08 (7)° and 59.41 (7)°, with the β-lactam, naphthalene and the mean plane of the xanthene ring system, respectively.

The molecular conformation is stabilized by two intramolecular C—H···O and C—H···N hydrogen contacts. Molecules are linked to each other by C—H···π interactions (Table 2 and Fig. 2).

### Experimental

A mixture of Schiff base *N*-(anthracen-9-ylmethylene)naphthalen-1-amine (0.3 g,1.45 mmol), triethylamine (0.73 g, 7.27 mmol), 9*H*-xanthen-9-carboxylic acid (0. 49 g, 2.18 mmol) and tosyl chloride (0.42 g, 2.18 mmol) in CH_2_Cl_2_(15 ml) was strirred at room temperature for 24 h. Then it was washed with 1 N HCl (20 ml) and saturated sodium bicarbonate solution (20 ml), brine (20 ml), dried (Na_2_SO_4_) and the solvent was evaporated to give the crude orange product which was then purified by recrystalization from C_6_H_14_ to result in orange prisms of (I) [yield 68%, m.p.: 457–459 K].

### Refinement

The H atoms were positioned geometrically and refined a riding model, with the C—H = 0.93–0.98 Å and with *U*_iso_(H) = 1.2*U*_eq_(C).

### Crystal data

**Table:**

C_39_H_25_NO_2_·0.5C_6_H_14_	*F*_000_ = 1228
*M**_r_* = 582.69	*D*_x_ = 1.275 Mg m^−^^3^
Monoclinic, *P*2_1_/*c*	Mo *K*α radiation λ = 0.71073 Å
Hall symbol: -P 2ybc	Cell parameters from 14824 reflections
*a* = 12.0788 (5) Å	θ = 1.4–28.1º
*b* = 14.1379 (5) Å	µ = 0.08 mm^−^^1^
*c* = 18.6133 (8) Å	*T* = 295 (2) K
β = 107.216 (3)º	Prism, orange
*V* = 3036.2 (2) Å^3^	0.55 × 0.38 × 0.26 mm
*Z* = 4	

### Data collection

**Table:**

Stoe IPDS2 diffractometer	6897 independent reflections
Monochromator: plane graphite	3841 reflections with *I* > 2σ(*I*)
Detector resolution: 6.67 pixels mm^-1^	*R*_int_ = 0.077
*T* = 295(2) K	θ_max_ = 27.5º
ω scans	θ_min_ = 1.8º
Absorption correction: integration(X-RED32; Stoe & Cie, 2002)	*h* = −15→15
*T*_min_ = 0.959, *T*_max_ = 0.980	*k* = −18→18
18377 measured reflections	*l* = −24→24

### Refinement

**Table:**

Refinement on *F*^2^	Secondary atom site location: difference Fourier map
Least-squares matrix: full	Hydrogen site location: inferred from neighbouring sites
*R*[*F*^2^ > 2σ(*F*^2^)] = 0.063	H-atom parameters constrained
*wR*(*F*^2^) = 0.191	*w* = 1/[σ^2^(*F*_o_^2^) + (0.1*P*)^2^], where *P* = (*F*_o_^2^ + 2*F*_c_^2^)/3
*S* = 0.97	(Δ/σ)_max_ < 0.001
6897 reflections	Δρ_max_ = 0.38 e Å^−^^3^
406 parameters	Δρ_min_ = −0.34 e Å^−^^3^
4 restraints	Extinction correction: none
Primary atom site location: structure-invariant direct methods	

### Special details

**Table:**

Geometry. Bond distances, angles *etc*. have been calculated using the rounded fractional coordinates. All su's are estimated from the variances of the (full) variance-covariance matrix. The cell e.s.d.'s are taken into account in the estimation of distances, angles and torsion angles
Refinement. Refinement on *F*^2^ for ALL reflections except those flagged by the user for potential systematic errors. Weighted *R*-factors *wR* and all goodnesses of fit *S* are based on *F*^2^, conventional *R*-factors *R* are based on *F*, with *F* set to zero for negative *F*^2^. The observed criterion of *F*^2^ > σ(*F*^2^) is used only for calculating -*R*-factor-obs *etc*. and is not relevant to the choice of reflections for refinement. *R*-factors based on *F*^2^ are statistically about twice as large as those based on *F*, and *R*-factors based on ALL data will be even larger.

### Fractional atomic coordinates and isotropic or equivalent isotropic displacement parameters (Å^2^)

**Table:**

	*x*	*y*	*z*	*U*_iso_*/*U*_eq_	
O1	0.21163 (13)	0.44035 (12)	0.32555 (9)	0.0596 (6)	
O2	0.56246 (17)	0.32446 (18)	0.51072 (19)	0.1023 (9)	
N1	0.13718 (15)	0.30979 (13)	0.37559 (10)	0.0486 (5)	
C1	0.21747 (17)	0.37492 (15)	0.36788 (12)	0.0475 (6)	
C2	0.31704 (18)	0.32796 (15)	0.42899 (13)	0.0494 (7)	
C3	0.22029 (18)	0.25267 (15)	0.43327 (12)	0.0484 (6)	
C4	0.19808 (18)	0.23182 (15)	0.50747 (12)	0.0473 (6)	
C5	0.10584 (18)	0.26912 (16)	0.53191 (12)	0.0491 (6)	
C6	0.0271 (2)	0.34110 (18)	0.49363 (14)	0.0603 (8)	
C7	−0.0628 (2)	0.3706 (2)	0.51804 (16)	0.0712 (9)	
C8	−0.0823 (2)	0.3328 (2)	0.58264 (16)	0.0766 (10)	
C9	−0.0094 (2)	0.2674 (2)	0.62223 (15)	0.0692 (9)	
C10	0.08760 (19)	0.23365 (17)	0.59976 (12)	0.0548 (7)	
C11	0.1614 (2)	0.16764 (19)	0.64230 (13)	0.0618 (8)	
C12	0.2579 (2)	0.13434 (17)	0.62269 (13)	0.0571 (7)	
C13	0.3353 (2)	0.0687 (2)	0.66867 (15)	0.0699 (9)	
C14	0.4275 (2)	0.0356 (2)	0.64914 (17)	0.0768 (10)	
C15	0.4477 (2)	0.0673 (2)	0.58261 (17)	0.0736 (10)	
C16	0.3756 (2)	0.13026 (18)	0.53649 (15)	0.0616 (8)	
C17	0.27561 (18)	0.16670 (16)	0.55416 (13)	0.0510 (7)	
C18	0.37023 (19)	0.38432 (17)	0.49830 (13)	0.0551 (7)	
C19	0.3051 (2)	0.4439 (2)	0.52930 (15)	0.0678 (9)	
C20	0.3522 (3)	0.4940 (2)	0.59465 (18)	0.0879 (11)	
C21	0.4670 (4)	0.4865 (3)	0.63036 (19)	0.1001 (15)	
C22	0.5360 (3)	0.4300 (3)	0.6015 (2)	0.0987 (14)	
C23	0.4880 (2)	0.3787 (2)	0.53559 (18)	0.0733 (9)	
C24	0.5246 (3)	0.2893 (2)	0.4393 (3)	0.0847 (13)	
C25	0.6081 (3)	0.2520 (3)	0.4083 (4)	0.127 (2)	
C26	0.5794 (5)	0.2166 (3)	0.3394 (5)	0.147 (3)	
C27	0.4646 (5)	0.2160 (3)	0.2944 (3)	0.124 (2)	
C28	0.3790 (3)	0.2518 (2)	0.32380 (19)	0.0884 (14)	
C29	0.4081 (2)	0.28880 (18)	0.39584 (16)	0.0647 (9)	
C30	0.02468 (18)	0.28109 (17)	0.33022 (12)	0.0516 (7)	
C31	0.0011 (2)	0.18699 (19)	0.32192 (15)	0.0648 (9)	
C32	−0.1085 (3)	0.1549 (2)	0.27798 (17)	0.0794 (11)	
C33	−0.1912 (2)	0.2177 (3)	0.24441 (17)	0.0784 (10)	
C34	−0.1725 (2)	0.3153 (2)	0.25397 (14)	0.0694 (9)	
C35	−0.2611 (2)	0.3819 (3)	0.2219 (2)	0.0932 (13)	
C36	−0.2437 (3)	0.4762 (3)	0.2340 (2)	0.1023 (15)	
C37	−0.1373 (2)	0.5103 (2)	0.27835 (19)	0.0825 (11)	
C38	−0.0490 (2)	0.44851 (19)	0.30912 (15)	0.0637 (8)	
C39	−0.06302 (19)	0.35014 (19)	0.29768 (12)	0.0564 (7)	
C40	0.0309 (4)	0.0054 (4)	0.4689 (3)	0.184 (4)	
C41	0.1171 (7)	−0.0660 (5)	0.5060 (4)	0.228 (5)	
C42	0.1800 (8)	−0.0640 (6)	0.4491 (7)	0.337 (8)	
H3	0.23460	0.19310	0.41060	0.0580*	
H6	0.03780	0.36860	0.45080	0.0720*	
H7	−0.11250	0.41700	0.49110	0.0850*	
H8	−0.14520	0.35280	0.59790	0.0920*	
H9	−0.02180	0.24310	0.66560	0.0830*	
H11	0.14710	0.14420	0.68540	0.0740*	
H13	0.32270	0.04800	0.71300	0.0840*	
H14	0.47740	−0.00810	0.67970	0.0920*	
H15	0.51170	0.04470	0.56990	0.0880*	
H16	0.39120	0.15010	0.49280	0.0740*	
H19	0.22630	0.45030	0.50480	0.0810*	
H20	0.30560	0.53260	0.61400	0.1060*	
H21	0.49940	0.51970	0.67470	0.1200*	
H22	0.61500	0.42590	0.62600	0.1190*	
H25	0.68560	0.25230	0.43690	0.1530*	
H26	0.63670	0.19170	0.32080	0.1760*	
H27	0.44510	0.19230	0.24570	0.1490*	
H28	0.30190	0.25070	0.29460	0.1060*	
H31	0.05780	0.14340	0.34550	0.0780*	
H32	−0.12350	0.09040	0.27220	0.0950*	
H33	−0.26240	0.19580	0.21420	0.0940*	
H35	−0.33280	0.36060	0.19200	0.1120*	
H36	−0.30330	0.51840	0.21240	0.1230*	
H37	−0.12630	0.57490	0.28700	0.0990*	
H38	0.02210	0.47190	0.33830	0.0760*	
H40A	−0.01690	−0.01330	0.41930	0.2200*	
H40B	0.06380	0.06770	0.46750	0.2200*	
H41A	0.08250	−0.12720	0.50920	0.2730*	
H41B	0.16480	−0.04620	0.55530	0.2730*	
H42A	0.24290	−0.10830	0.46270	0.5050*	
H42B	0.20990	−0.00150	0.44670	0.5050*	
H42C	0.12800	−0.08070	0.40090	0.5050*	

### Atomic displacement parameters (Å^2^)

**Table:**

	*U*^11^	*U*^22^	*U*^33^	*U*^12^	*U*^13^	*U*^23^
O1	0.0536 (9)	0.0634 (10)	0.0609 (10)	−0.0002 (7)	0.0158 (7)	0.0146 (8)
O2	0.0468 (11)	0.0959 (16)	0.150 (2)	0.0110 (10)	0.0071 (13)	0.0133 (16)
N1	0.0468 (9)	0.0546 (10)	0.0439 (9)	−0.0002 (7)	0.0129 (7)	0.0018 (8)
C1	0.0463 (11)	0.0527 (12)	0.0447 (10)	0.0017 (9)	0.0155 (9)	0.0026 (10)
C2	0.0436 (11)	0.0535 (12)	0.0539 (12)	0.0055 (9)	0.0189 (9)	0.0066 (10)
C3	0.0511 (11)	0.0498 (11)	0.0466 (11)	0.0030 (9)	0.0181 (9)	0.0015 (9)
C4	0.0488 (11)	0.0472 (11)	0.0464 (11)	−0.0014 (9)	0.0151 (9)	−0.0012 (9)
C5	0.0489 (11)	0.0543 (12)	0.0434 (10)	−0.0036 (9)	0.0125 (9)	−0.0047 (10)
C6	0.0658 (14)	0.0656 (14)	0.0530 (12)	0.0109 (11)	0.0232 (11)	−0.0015 (11)
C7	0.0691 (16)	0.0813 (18)	0.0656 (15)	0.0170 (14)	0.0237 (13)	−0.0047 (14)
C8	0.0684 (16)	0.099 (2)	0.0720 (17)	0.0091 (15)	0.0356 (14)	−0.0082 (16)
C9	0.0681 (15)	0.0899 (19)	0.0597 (14)	−0.0039 (14)	0.0344 (13)	−0.0037 (14)
C10	0.0527 (12)	0.0649 (14)	0.0477 (12)	−0.0080 (10)	0.0163 (10)	−0.0041 (11)
C11	0.0630 (14)	0.0772 (16)	0.0465 (12)	−0.0068 (12)	0.0182 (11)	0.0036 (12)
C12	0.0566 (13)	0.0602 (13)	0.0485 (12)	−0.0065 (10)	0.0062 (10)	0.0042 (11)
C13	0.0678 (15)	0.0760 (17)	0.0581 (14)	0.0004 (13)	0.0065 (12)	0.0148 (13)
C14	0.0660 (16)	0.0768 (18)	0.0745 (18)	0.0096 (13)	0.0008 (13)	0.0178 (15)
C15	0.0572 (14)	0.0766 (18)	0.0805 (18)	0.0144 (12)	0.0105 (13)	0.0041 (15)
C16	0.0576 (13)	0.0653 (14)	0.0631 (14)	0.0063 (11)	0.0199 (11)	0.0072 (12)
C17	0.0475 (11)	0.0514 (12)	0.0518 (12)	−0.0042 (9)	0.0113 (9)	−0.0002 (10)
C18	0.0459 (11)	0.0608 (13)	0.0548 (12)	−0.0044 (10)	0.0091 (10)	0.0104 (11)
C19	0.0621 (14)	0.0777 (17)	0.0620 (15)	−0.0107 (12)	0.0160 (12)	−0.0105 (13)
C20	0.100 (2)	0.091 (2)	0.0703 (18)	−0.0270 (18)	0.0215 (18)	−0.0175 (16)
C21	0.121 (3)	0.100 (3)	0.0594 (17)	−0.038 (2)	−0.0038 (19)	0.0046 (18)
C22	0.0737 (19)	0.099 (3)	0.090 (2)	−0.0298 (19)	−0.0274 (18)	0.034 (2)
C23	0.0497 (13)	0.0725 (16)	0.0859 (18)	−0.0034 (12)	0.0017 (13)	0.0227 (15)
C24	0.0568 (16)	0.0672 (17)	0.143 (3)	0.0137 (13)	0.0493 (19)	0.028 (2)
C25	0.079 (2)	0.087 (3)	0.248 (6)	0.0196 (19)	0.099 (4)	0.023 (3)
C26	0.150 (4)	0.087 (3)	0.266 (7)	0.035 (3)	0.159 (5)	0.030 (4)
C27	0.191 (5)	0.083 (2)	0.151 (4)	0.040 (3)	0.131 (4)	0.019 (2)
C28	0.120 (3)	0.0764 (19)	0.092 (2)	0.0296 (17)	0.067 (2)	0.0136 (17)
C29	0.0645 (15)	0.0584 (14)	0.0864 (18)	0.0164 (11)	0.0457 (14)	0.0202 (13)
C30	0.0497 (11)	0.0655 (14)	0.0422 (10)	−0.0086 (10)	0.0177 (9)	−0.0053 (10)
C31	0.0659 (15)	0.0667 (15)	0.0646 (15)	−0.0117 (12)	0.0237 (12)	−0.0070 (13)
C32	0.085 (2)	0.0829 (19)	0.0791 (18)	−0.0320 (17)	0.0381 (17)	−0.0203 (16)
C33	0.0557 (15)	0.109 (2)	0.0729 (17)	−0.0250 (16)	0.0227 (13)	−0.0224 (17)
C34	0.0469 (13)	0.103 (2)	0.0591 (14)	−0.0113 (13)	0.0171 (11)	−0.0092 (14)
C35	0.0448 (14)	0.130 (3)	0.094 (2)	0.0001 (16)	0.0039 (14)	−0.001 (2)
C36	0.0560 (18)	0.119 (3)	0.120 (3)	0.0200 (18)	0.0078 (18)	0.013 (2)
C37	0.0563 (15)	0.087 (2)	0.100 (2)	0.0097 (14)	0.0168 (15)	0.0100 (17)
C38	0.0501 (12)	0.0719 (16)	0.0661 (15)	−0.0005 (11)	0.0125 (11)	0.0034 (13)
C39	0.0468 (11)	0.0779 (16)	0.0458 (11)	−0.0050 (11)	0.0156 (9)	−0.0043 (11)
C40	0.082 (4)	0.103 (3)	0.304 (12)	−0.005 (3)	−0.038 (4)	−0.075 (5)
C41	0.289 (10)	0.270 (10)	0.162 (5)	−0.219 (9)	0.126 (7)	−0.125 (6)
C42	0.307 (13)	0.164 (7)	0.44 (2)	−0.106 (8)	−0.042 (13)	−0.011 (10)

### Geometric parameters (Å, °)

**Table:**

O1—C1	1.204 (3)	C33—C34	1.401 (5)
O2—C23	1.363 (4)	C34—C35	1.418 (4)
O2—C24	1.365 (6)	C34—C39	1.420 (3)
N1—C1	1.375 (3)	C35—C36	1.358 (6)
N1—C3	1.475 (3)	C36—C37	1.392 (5)
N1—C30	1.430 (3)	C37—C38	1.366 (4)
C1—C2	1.540 (3)	C38—C39	1.410 (4)
C2—C3	1.600 (3)	C3—H3	0.9800
C2—C18	1.490 (3)	C6—H6	0.9300
C2—C29	1.516 (3)	C7—H7	0.9300
C3—C4	1.512 (3)	C8—H8	0.9300
C4—C5	1.424 (3)	C9—H9	0.9300
C4—C17	1.414 (3)	C11—H11	0.9300
C5—C6	1.431 (3)	C13—H13	0.9300
C5—C10	1.435 (3)	C14—H14	0.9300
C6—C7	1.361 (4)	C15—H15	0.9300
C7—C8	1.398 (4)	C16—H16	0.9300
C8—C9	1.338 (4)	C19—H19	0.9300
C9—C10	1.437 (4)	C20—H20	0.9300
C10—C11	1.369 (3)	C21—H21	0.9300
C11—C12	1.402 (4)	C22—H22	0.9300
C12—C13	1.412 (4)	C25—H25	0.9300
C12—C17	1.430 (3)	C26—H26	0.9300
C13—C14	1.353 (4)	C27—H27	0.9300
C14—C15	1.405 (4)	C28—H28	0.9300
C15—C16	1.358 (4)	C31—H31	0.9300
C16—C17	1.438 (3)	C32—H32	0.9300
C18—C19	1.389 (4)	C33—H33	0.9300
C18—C23	1.388 (4)	C35—H35	0.9300
C19—C20	1.377 (4)	C36—H36	0.9300
C20—C21	1.353 (6)	C37—H37	0.9300
C21—C22	1.373 (6)	C38—H38	0.9300
C22—C23	1.395 (5)	C40—C41	1.468 (9)
C24—C25	1.405 (6)	C40—C40^i^	1.560 (7)
C24—C29	1.400 (5)	C41—C42	1.475 (14)
C25—C26	1.324 (11)	C40—H40A	0.9700
C26—C27	1.392 (10)	C40—H40B	0.9700
C27—C28	1.400 (7)	C41—H41A	0.9700
C28—C29	1.384 (4)	C41—H41B	0.9700
C30—C31	1.360 (4)	C42—H42A	0.9600
C30—C39	1.436 (3)	C42—H42B	0.9600
C31—C32	1.409 (4)	C42—H42C	0.9600
C32—C33	1.344 (5)		
			
O1···C28	3.352 (4)	C33···H9^iv^	2.9000
O1···C38	3.074 (3)	C34···H9^iv^	2.9100
O1···H38	2.4100	C38···H6	2.7700
O1···H32^ii^	2.7900	C39···H6	2.7600
O1···H22^iii^	2.7700	C39···H9^iv^	2.9600
O1···H11^iv^	2.7600	H3···C16	2.6100
O1···H13^iv^	2.8100	H3···H31	2.2300
N1···C6	2.919 (3)	H3···H16	2.1400
N1···C28	3.435 (4)	H3···C31	2.8200
N1···H6	2.2600	H3···C28	2.8300
N1···H38	2.6600	H6···C3	2.8400
C1···C38	3.249 (3)	H6···C1	3.0100
C2···C16	3.388 (3)	H6···C39	2.7600
C4···C19	3.243 (4)	H6···C30	2.5300
C5···C30	3.592 (3)	H6···C38	2.7700
C5···C19	3.460 (4)	H6···N1	2.2600
C6···C39	3.486 (3)	H6···H19	2.4800
C6···C30	3.150 (3)	H6···H38	2.5100
C6···N1	2.919 (3)	H7···H19^vi^	2.3400
C6···C19	3.537 (4)	H7···C19^vi^	2.9800
C9···C42^i^	3.558 (10)	H9···H11	2.4100
C10···C41^i^	3.567 (8)	H9···C30^vii^	2.9700
C11···C40^i^	3.580 (6)	H9···C31^vii^	3.0100
C14···C26^v^	3.574 (5)	H9···C39^vii^	2.9600
C16···C29	3.555 (4)	H9···C34^vii^	2.9100
C16···C2	3.388 (3)	H9···C32^vii^	2.9700
C17···C18	3.544 (3)	H9···C33^vii^	2.9000
C18···C22^iii^	3.588 (5)	H11···H9	2.4100
C18···C17	3.544 (3)	H11···H13	2.4400
C19···C6	3.537 (4)	H11···O1^vii^	2.7600
C19···C5	3.460 (4)	H13···H11	2.4400
C19···C4	3.243 (4)	H13···H21^viii^	2.5500
C20···C24^iii^	3.544 (4)	H13···O1^vii^	2.8100
C21···C24^iii^	3.438 (5)	H14···C27^v^	3.0300
C22···C18^iii^	3.588 (5)	H14···C26^v^	3.0200
C24···C20^iii^	3.544 (4)	H14···H36^ix^	2.5400
C24···C21^iii^	3.438 (5)	H16···C2	2.8100
C26···C14^v^	3.574 (5)	H16···C3	2.5000
C28···N1	3.435 (4)	H16···H3	2.1400
C28···O1	3.352 (4)	H16···C29	2.7100
C29···C16	3.555 (4)	H16···C24	2.9000
C30···C6	3.150 (3)	H19···C5	3.0600
C30···C5	3.592 (3)	H19···C3	3.0900
C38···C1	3.249 (3)	H19···H7^vi^	2.3400
C38···O1	3.074 (3)	H19···C6	2.8100
C39···C6	3.486 (3)	H19···H6	2.4800
C40···C11^i^	3.580 (6)	H19···C1	2.7400
C41···C10^i^	3.567 (8)	H21···C13^xi^	3.0900
C42···C9^i^	3.558 (10)	H21···H13^xi^	2.5500
C1···H28	2.6100	H22···H33^ix^	2.5300
C1···H19	2.7400	H22···O1^iii^	2.7700
C1···H6	3.0100	H26···C33^xii^	2.8700
C1···H38	2.6400	H28···C1	2.6100
C2···H16	2.8100	H28···C3	3.0200
C3···H28	3.0200	H28···C11^iv^	3.0700
C3···H31	2.6500	H31···H40B	2.4900
C3···H6	2.8400	H31···H3	2.2300
C3···H16	2.5000	H31···C3	2.6500
C3···H19	3.0900	H32···O1^xiii^	2.7900
C4···H40B	2.8000	H33···H35	2.4700
C5···H41A^i^	2.9600	H33···H22^xiv^	2.5300
C5···H19	3.0600	H35···H33	2.4700
C5···H40B	3.0700	H35···C15^xiv^	3.0000
C6···H19	2.8100	H36···H14^xiv^	2.5400
C9···H42C^i^	2.9700	H37···C9^vi^	2.9800
C9···H37^vi^	2.9800	H38···O1	2.4100
C9···H41A^i^	3.0700	H38···N1	2.6600
C10···H41A^i^	2.8500	H38···C1	2.6400
C11···H40A^i^	2.8200	H38···H6	2.5100
C11···H28^vii^	3.0700	H40A···H42C	2.1100
C12···H41B	2.9200	H40A···C11^i^	2.8200
C13···H21^viii^	3.0900	H40A···H41B^i^	2.1500
C13···H41B	2.9600	H40B···C4	2.8000
C15···H35^ix^	3.0000	H40B···C5	3.0700
C16···H3	2.6100	H40B···C17	2.9400
C16···H42B	2.8800	H40B···C31	3.0900
C17···H40B	2.9400	H40B···H31	2.4900
C17···H42B	3.0600	H40B···H42B	2.1500
C19···H7^vi^	2.9800	H40B···H41A^i^	2.1100
C24···H16	2.9000	H41A···C5^i^	2.9600
C26···H14^v^	3.0200	H41A···C9^i^	3.0700
C27···H14^v^	3.0300	H41A···C10^i^	2.8500
C28···H3	2.8300	H41A···H40B^i^	2.1100
C29···H16	2.7100	H41B···C12	2.9200
C30···H6	2.5300	H41B···C13	2.9600
C30···H9^iv^	2.9700	H41B···H40A^i^	2.1500
C31···H40B	3.0900	H42B···C16	2.8800
C31···H9^iv^	3.0100	H42B···C17	3.0600
C31···H3	2.8200	H42B···H40B	2.1500
C32···H9^iv^	2.9700	H42C···H40A	2.1100
C33···H26^x^	2.8700	H42C···C9^i^	2.9700
			
C23—O2—C24	118.1 (3)	C30—C39—C34	116.8 (2)
C1—N1—C3	95.37 (17)	C30—C39—C38	124.5 (2)
C1—N1—C30	134.79 (19)	C34—C39—C38	118.6 (2)
C3—N1—C30	126.70 (18)	N1—C3—H3	109.00
O1—C1—N1	132.6 (2)	C2—C3—H3	109.00
O1—C1—C2	134.2 (2)	C4—C3—H3	109.00
N1—C1—C2	93.15 (17)	C5—C6—H6	119.00
C1—C2—C3	84.34 (16)	C7—C6—H6	119.00
C1—C2—C18	116.82 (18)	C6—C7—H7	119.00
C1—C2—C29	111.03 (19)	C8—C7—H7	119.00
C3—C2—C18	116.53 (19)	C7—C8—H8	120.00
C3—C2—C29	113.94 (18)	C9—C8—H8	120.00
C18—C2—C29	111.6 (2)	C8—C9—H9	119.00
N1—C3—C2	87.09 (15)	C10—C9—H9	119.00
N1—C3—C4	120.35 (19)	C10—C11—H11	119.00
C2—C3—C4	120.32 (18)	C12—C11—H11	119.00
C3—C4—C5	125.94 (19)	C12—C13—H13	119.00
C3—C4—C17	115.10 (19)	C14—C13—H13	120.00
C5—C4—C17	118.9 (2)	C13—C14—H14	120.00
C4—C5—C6	124.9 (2)	C15—C14—H14	120.00
C4—C5—C10	119.4 (2)	C14—C15—H15	119.00
C6—C5—C10	115.7 (2)	C16—C15—H15	119.00
C5—C6—C7	122.0 (2)	C15—C16—H16	119.00
C6—C7—C8	121.6 (3)	C17—C16—H16	120.00
C7—C8—C9	119.2 (2)	C18—C19—H19	119.00
C8—C9—C10	122.0 (2)	C20—C19—H19	119.00
C5—C10—C9	119.4 (2)	C19—C20—H20	120.00
C5—C10—C11	120.3 (2)	C21—C20—H20	120.00
C9—C10—C11	120.4 (2)	C20—C21—H21	120.00
C10—C11—C12	121.8 (2)	C22—C21—H21	120.00
C11—C12—C13	121.0 (2)	C21—C22—H22	120.00
C11—C12—C17	118.7 (2)	C23—C22—H22	120.00
C13—C12—C17	120.3 (2)	C24—C25—H25	119.00
C12—C13—C14	121.0 (2)	C26—C25—H25	119.00
C13—C14—C15	119.9 (3)	C25—C26—H26	120.00
C14—C15—C16	121.3 (2)	C27—C26—H26	120.00
C15—C16—C17	121.0 (2)	C26—C27—H27	121.00
C4—C17—C12	120.7 (2)	C28—C27—H27	121.00
C4—C17—C16	122.8 (2)	C27—C28—H28	120.00
C12—C17—C16	116.6 (2)	C29—C28—H28	120.00
C2—C18—C19	122.1 (2)	C30—C31—H31	120.00
C2—C18—C23	121.2 (2)	C32—C31—H31	120.00
C19—C18—C23	116.7 (2)	C31—C32—H32	120.00
C18—C19—C20	122.8 (3)	C33—C32—H32	120.00
C19—C20—C21	119.3 (3)	C32—C33—H33	119.00
C20—C21—C22	120.4 (3)	C34—C33—H33	119.00
C21—C22—C23	120.3 (3)	C34—C35—H35	119.00
O2—C23—C18	123.0 (3)	C36—C35—H35	119.00
O2—C23—C22	116.6 (3)	C35—C36—H36	120.00
C18—C23—C22	120.5 (3)	C37—C36—H36	120.00
O2—C24—C25	117.6 (4)	C36—C37—H37	120.00
O2—C24—C29	123.7 (3)	C38—C37—H37	120.00
C25—C24—C29	118.8 (5)	C37—C38—H38	119.00
C24—C25—C26	121.7 (5)	C39—C38—H38	119.00
C25—C26—C27	120.9 (6)	C40^i^—C40—C41	90.7 (5)
C26—C27—C28	118.8 (5)	C40—C41—C42	95.4 (6)
C27—C28—C29	120.7 (4)	C41—C40—H40A	114.00
C2—C29—C24	119.3 (3)	C41—C40—H40B	113.00
C2—C29—C28	121.7 (3)	H40A—C40—H40B	111.00
C24—C29—C28	119.0 (3)	C40^i^—C40—H40A	114.00
N1—C30—C31	118.4 (2)	C40^i^—C40—H40B	114.00
N1—C30—C39	120.6 (2)	C40—C41—H41A	113.00
C31—C30—C39	120.9 (2)	C40—C41—H41B	113.00
C30—C31—C32	120.7 (2)	C42—C41—H41A	113.00
C31—C32—C33	119.9 (3)	C42—C41—H41B	113.00
C32—C33—C34	121.5 (3)	H41A—C41—H41B	110.00
C33—C34—C35	121.8 (3)	C41—C42—H42A	110.00
C33—C34—C39	120.2 (2)	C41—C42—H42B	109.00
C35—C34—C39	118.0 (3)	C41—C42—H42C	109.00
C34—C35—C36	121.4 (3)	H42A—C42—H42B	109.00
C35—C36—C37	120.6 (3)	H42A—C42—H42C	110.00
C36—C37—C38	119.8 (3)	H42B—C42—H42C	109.00
C37—C38—C39	121.6 (2)		
			
C24—O2—C23—C18	13.2 (4)	C8—C9—C10—C11	179.0 (3)
C24—O2—C23—C22	−166.5 (3)	C5—C10—C11—C12	1.6 (4)
C23—O2—C24—C25	166.1 (3)	C9—C10—C11—C12	−178.9 (2)
C23—O2—C24—C29	−13.7 (5)	C10—C11—C12—C13	177.8 (2)
C30—N1—C1—O1	14.4 (4)	C10—C11—C12—C17	−3.4 (4)
C3—N1—C1—C2	−1.87 (17)	C13—C12—C17—C4	179.5 (2)
C3—N1—C1—O1	174.3 (3)	C13—C12—C17—C16	−1.0 (3)
C30—N1—C3—C2	164.1 (2)	C11—C12—C13—C14	179.1 (3)
C1—N1—C3—C4	125.6 (2)	C11—C12—C17—C16	−179.8 (2)
C30—N1—C3—C4	−72.1 (3)	C17—C12—C13—C14	0.3 (4)
C1—N1—C3—C2	1.79 (17)	C11—C12—C17—C4	0.7 (3)
C30—N1—C1—C2	−161.8 (2)	C12—C13—C14—C15	0.6 (4)
C1—N1—C30—C39	−48.7 (3)	C13—C14—C15—C16	−0.7 (4)
C1—N1—C30—C31	134.7 (3)	C14—C15—C16—C17	−0.1 (4)
C3—N1—C30—C39	156.6 (2)	C15—C16—C17—C4	−179.6 (2)
C3—N1—C30—C31	−20.1 (3)	C15—C16—C17—C12	0.9 (4)
N1—C1—C2—C29	115.22 (19)	C2—C18—C23—O2	2.0 (4)
N1—C1—C2—C3	1.72 (16)	C23—C18—C19—C20	−1.5 (4)
N1—C1—C2—C18	−115.2 (2)	C2—C18—C23—C22	−178.3 (3)
O1—C1—C2—C18	68.8 (3)	C19—C18—C23—O2	−178.8 (3)
O1—C1—C2—C29	−60.8 (3)	C19—C18—C23—C22	1.0 (4)
O1—C1—C2—C3	−174.3 (3)	C2—C18—C19—C20	177.8 (3)
C1—C2—C18—C23	−144.4 (2)	C18—C19—C20—C21	0.8 (5)
C1—C2—C29—C28	−33.8 (3)	C19—C20—C21—C22	0.4 (5)
C1—C2—C3—C4	−125.4 (2)	C20—C21—C22—C23	−0.9 (6)
C1—C2—C3—N1	−1.60 (15)	C21—C22—C23—C18	0.2 (5)
C18—C2—C3—N1	115.6 (2)	C21—C22—C23—O2	179.9 (3)
C3—C2—C29—C24	−120.1 (3)	C29—C24—C25—C26	0.0 (6)
C3—C2—C29—C28	59.4 (3)	O2—C24—C29—C2	−0.8 (4)
C1—C2—C29—C24	146.7 (2)	O2—C24—C29—C28	179.7 (3)
C29—C2—C3—C4	124.1 (2)	O2—C24—C25—C26	−179.8 (4)
C1—C2—C18—C19	36.3 (3)	C25—C24—C29—C2	179.4 (3)
C18—C2—C3—C4	−8.2 (3)	C25—C24—C29—C28	−0.1 (5)
C18—C2—C29—C24	14.5 (3)	C24—C25—C26—C27	0.8 (8)
C18—C2—C29—C28	−166.1 (2)	C25—C26—C27—C28	−1.3 (7)
C29—C2—C18—C23	−15.2 (3)	C26—C27—C28—C29	1.2 (6)
C29—C2—C3—N1	−112.1 (2)	C27—C28—C29—C2	−180.0 (3)
C29—C2—C18—C19	165.6 (2)	C27—C28—C29—C24	−0.5 (5)
C3—C2—C18—C23	118.2 (3)	N1—C30—C31—C32	179.8 (2)
C3—C2—C18—C19	−61.1 (3)	C39—C30—C31—C32	3.2 (4)
C2—C3—C4—C17	−81.8 (2)	N1—C30—C39—C34	−179.5 (2)
N1—C3—C4—C5	−5.6 (3)	N1—C30—C39—C38	−2.5 (3)
N1—C3—C4—C17	172.24 (19)	C31—C30—C39—C34	−2.9 (3)
C2—C3—C4—C5	100.3 (3)	C31—C30—C39—C38	174.1 (2)
C3—C4—C17—C16	6.3 (3)	C30—C31—C32—C33	−0.6 (4)
C17—C4—C5—C10	−5.5 (3)	C31—C32—C33—C34	−2.1 (5)
C3—C4—C17—C12	−174.3 (2)	C32—C33—C34—C35	−176.8 (3)
C3—C4—C5—C6	−7.7 (4)	C32—C33—C34—C39	2.3 (4)
C5—C4—C17—C12	3.7 (3)	C33—C34—C35—C36	177.3 (3)
C17—C4—C5—C6	174.5 (2)	C39—C34—C35—C36	−1.8 (4)
C5—C4—C17—C16	−175.7 (2)	C33—C34—C39—C30	0.2 (3)
C3—C4—C5—C10	172.3 (2)	C33—C34—C39—C38	−177.0 (2)
C6—C5—C10—C9	3.5 (3)	C35—C34—C39—C30	179.3 (2)
C6—C5—C10—C11	−177.1 (2)	C35—C34—C39—C38	2.1 (4)
C4—C5—C10—C11	2.9 (3)	C34—C35—C36—C37	0.3 (5)
C4—C5—C6—C7	176.8 (2)	C35—C36—C37—C38	1.0 (5)
C10—C5—C6—C7	−3.2 (4)	C36—C37—C38—C39	−0.6 (4)
C4—C5—C10—C9	−176.5 (2)	C37—C38—C39—C30	−177.9 (3)
C5—C6—C7—C8	0.8 (4)	C37—C38—C39—C34	−1.0 (4)
C6—C7—C8—C9	1.4 (4)	C40^i^—C40—C41—C42	178.2 (6)
C7—C8—C9—C10	−1.0 (4)	C41—C40—C40^i^—C41^i^	180.0 (5)
C8—C9—C10—C5	−1.5 (4)		

Symmetry codes: (i) −*x*, −*y*, −*z*+1; (ii) −*x*, *y*+1/2, −*z*+1/2; (iii) −*x*+1, −*y*+1, −*z*+1; (iv) *x*, −*y*+1/2, *z*−1/2; (v) −*x*+1, −*y*, −*z*+1; (vi) −*x*, −*y*+1, −*z*+1; (vii) *x*, −*y*+1/2, *z*+1/2; (viii) −*x*+1, *y*−1/2, −*z*+3/2; (ix) *x*+1, −*y*+1/2, *z*+1/2; (x) *x*−1, *y*, *z*; (xi) −*x*+1, *y*+1/2, −*z*+3/2; (xii) *x*+1, *y*, *z*; (xiii) −*x*, *y*−1/2, −*z*+1/2; (xiv) *x*−1, −*y*+1/2, *z*−1/2.

### Hydrogen-bond geometry (Å, °)

**Table:**

*D*—H···*A*	*D*—H	H···*A*	*D*···*A*	*D*—H···*A*
C6—H6···N1	0.93	2.26	2.919 (3)	128
C38—H38···O1	0.93	2.41	3.074 (3)	128
C9—H9···Cg3^vii^	0.93	2.60	3.471 (3)	156
C40—H40B···Cg1	0.97	2.82	3.572 (6)	135
C41—H41A···Cg2^i^	0.97	2.85	3.728 (7)	150

Symmetry codes: (vii) *x*, −*y*+1/2, *z*+1/2;  (i) −*x*, −*y*, −*z*+1.
